# Surgical management of tumor thrombus into the right atrium

**DOI:** 10.34172/jcvtr.2023.31597

**Published:** 2023-06-29

**Authors:** Beatriz Acuña Pais, Rocío Casais, Julio Lugo, Miguel Á. Piñón, Juan. J. Legarra

**Affiliations:** Cardiovascular Surgery Service, Álvaro Cunqueiro Hospital, Carretera de Clara Campoamor, Vigo, Spain

**Keywords:** Cardiopulmonary Bypass, Tumor Thrombus, Right Atrium

## Abstract

Renal cell carcinoma represents 3% of solid tumors. In 4-10% of patients, venous tumor thrombosis is present, and 1% reaches the right atrium. Other tumors can be associated with tumor thrombosis. The natural history of venous tumor thrombosis implies a mean survival of 5 months. Between 2001 and 2021, 4 patients underwent resection of tumor thrombi into the right atrium, requiring cardiopulmonary bypass. None of the patients died within 30 days postoperatively. Mean follow-up time was 30.9 months (2.4- 96.1). Two patients are still alive and two died due to disease progression. Died patient’s follow-up was 7.5 and 17.4 months, surpassing life expectancy of those without surgery. We present a series of patients who underwent lumpectomy and IVC thrombectomy. IVC tumor thrombosis has an ominous prognosis, however surgical treatment has an important role by improving the survival of these patients. The multidisciplinary approach is necessary to obtain good postoperative results.

## Introduction

Renal cell carcinoma (RCC) represents 3% of solid tumors.^1^ In 4-10% of patients, RCC grows intraluminal towards the renal venous system, in a cranial direction, producing a tumoral venous thrombosis that reaches the right atrium in 1% of cases.^1,2^ Other abdominal tumors can be associated with IVC tumor thrombosis, such as adrenal carcinoma, leiomyosarcoma of the cava, paratesticular rhabdomyosarcoma, hepatocarcinoma, pheochromocytoma, and Willms tumor.^3^ The diagnosis of this complication is made by ultrasound or incidental finding in the preoperative CT scan. Many patients do not present symptoms of tumor thrombus, but its presence should be suspected in case of lower limbs edema, dilatation of superficial abdominal veins, proteinuria, pulmonary embolism, right varicocele and right atrial mass.^1^ The natural history of tumoral venous thrombosis due to RCC presents a mean survival of 5 months, and survival of 29% in a year.^4^

Between 45-70% of patients can benefit from tumor thrombus resection surgery and nephrectomy.^2^ The greater the extension of the thrombus the worsen surgical morbidity and mortality: when the thrombus exceeds the possibility of clamping the inferior vena cava (IVC) at its infrahepatic limit, clamping of the intrahepatic cava and the hepatic pedicle, and veno-venous bypass or the use of extracorporeal circulation (ECC) is required.^3^ This approach has been used for patients with other types of tumors with thrombus in the inferior vena cava (IVC).^5^

 We describe our experience in the surgical management of patients with tumor thrombi invading IVC.

 The objective of this work is to determine the role of ECC in the treatment of tumor thrombi that extend to the supradiaphragmatic IVC.

## Description of the cases

 Between 2001 and 2021, 4 patients with tumor of renal, adrenal and hepatic origin with thrombus into the right atrium underwent surgery at our center. To carry out the resection, all of them required hemodynamic support with cardiopulmonary bypass (CPB).

 Once informed consent of the patients was obtained following the requisites of the Local Ethics Committee; demographic, operative and postoperative data were collected.

**Figure 1:**
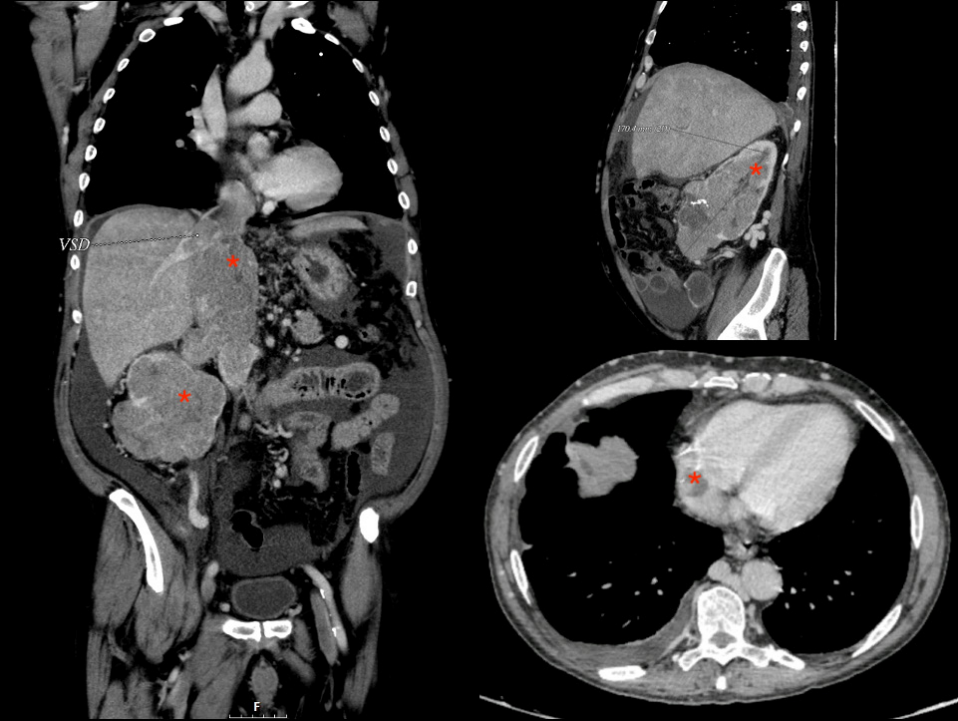
Images of the preoperative CT of one of the patients. Pathological areas are marked with an asterisk

To determine the extent of the tumor, we used CT or MRI images, and performed an echocardiogram and preoperative coronary angiography depending on the indication. Figure 1 shows preoperative CT cuts of one of the patients. Tumor staging was based on the results of the different complementary tests; and the types of IVC tumor thrombi in the case of RCC and adrenal cell carcinoma were assigned according to their specific classifications.^6,7^ Table 1 shows the Mayo classification for tumor thrombi.^6^

**Table 1:** Mayo classification for RCC tumor thrombi of the inferior vena cava

**Tumor thrombus level**	**Definition**
0	Tumor thrombus limited to the renal vein
I	Invasive tumor thrombus of the IVC less than 2cm from the renal vein
II	Invasive tumor thrombus of the IVC more than 2cm from the renal vein but infrahepatic.
III	Tumor thrombus suprahepatic but infradiaphragmatic
IV	Supradiaphragmatic tumor thrombus, including atrial thrombus

Inferior vena cava: (IVC), Renal cell carcinoma (RCC)

 The multidisciplinary team compound of urologists, general surgeons, vascular surgeons, anesthesiologists and cardiovascular surgeons was in charge of preparing an individualized surgical plan for each patient.

**Figure 2:**
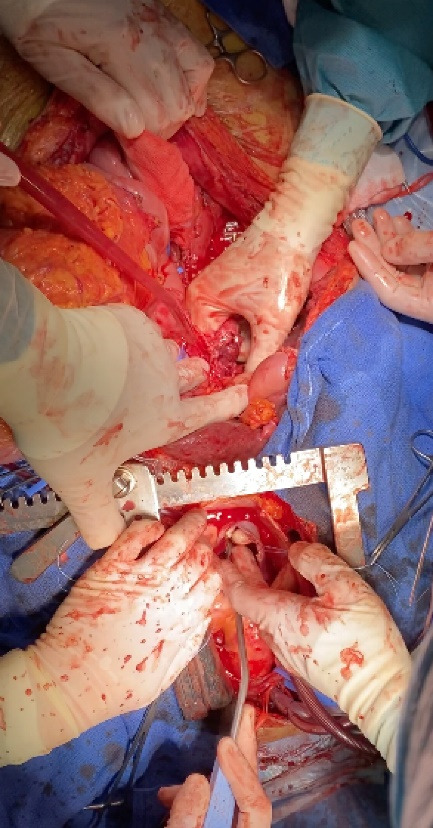
Incisions used to perform the intervention

**Figure 3:**
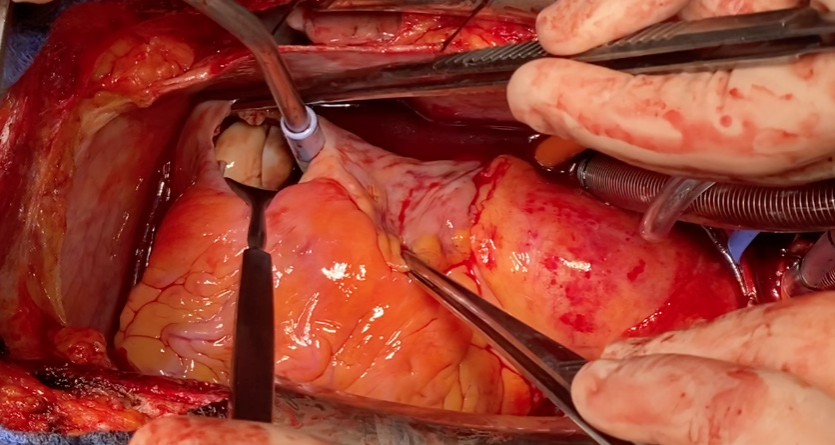
Proximal portion of the tumor thrombus in the right atrium

**Figure 4:**
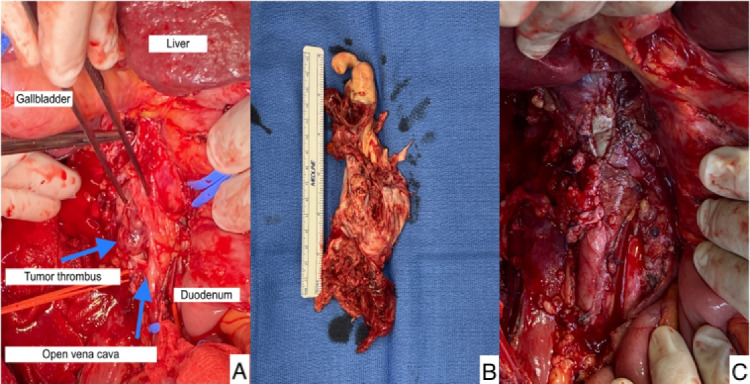
**A.** IVC with the thrombus inside **B.** Tumor thrombus after extraction **C.** IVC reconstruction with a bovine pericardial patch after thrombectomy

Below we describe the tumor thrombectomy technique according to the intervention performed on patient 4. The intervention consisted of ultrasound-guided puncture of the left common femoral vein and insertion of a 7Fr introducer sheath. Through a laparotomy, the intestines were oriented to dissect the peritoneum and expose the retroperitoneal space. Standard nephrectomy was performed without ligation of the corresponding renal vein. Using the Pringle maneuver, the liver was mobilized to release the retrohepatic vena cava. After median sternotomy and systemic heparinization we performed for cannulation of the superior vena cava (SVC), common femoral vein (CFV) and brachiocephalic trunk (BCT), and started ECC. Figure 2 shows the incisions used to perform the intervention. The SVC was excluded and a right atriotomy was performed. Figure 3 shows the proximal portion of the tumor thrombus in the right atrium. In turn, the IVC was opened and a thrombectomy was performed, in a caudal direction to the right atrium, extracting the piece en bloc. Figure 4 shows the IVC with the thrombus inside.

 In two of the reported cases, the tumor thrombus had significant adhesions to the wall of the vena cava, requiring vessel reconstruction with a bovine pericardial patch after thrombectomy. Closure of the cavotomy is the last surgical step prior to disconnection of the ECC.

**Figure 5:**
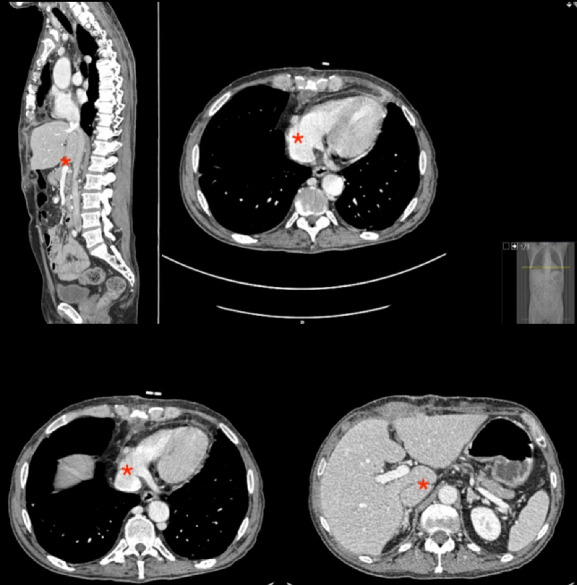
Images from the postoperative CT scan of one of the patients, demonstrating the absence of thrombus. The regressed regions are marked with an asterisk

The postoperative period of the patients consisted of a short stay in the Intensive Care Unit and subsequent transfer to the ward where a control CT scan was performed prior to discharge. Figure 5 shows cuts of the postoperative CT of one of the patients, demonstrating the absence of thrombus.

## Results

Between 2001 and 2021, four patients underwent consecutive surgery in our center. The demographic and clinicopathological characteristics are detailed in Table 2. All of them presented optimal preoperative conditions. At the time of surgery, the median age was 59 years (43-68). Two patients had adrenal cell carcinoma, one hepatocarcinoma, and one clear cell renal carcinoma. One of them had metastatic disease before the intervention. All patients had tumor thrombus into the right atrium. When deciding the treatment of tumor thrombus, we reviewed the personal history of the patients in order to take on account conditions like heart failure or dialysis treatment that could threaten surgical outcomes. In spite that the four patients were active smokers, none of them had other personal history conditions, therefore they were suitable for intervention. None of them received preoperative radio-chemotherapy. In patient 3, surgeons checked the absence of bland thrombus below the tumor thrombus since it is a contraindication to use veno-venous bypass. In order to reduce intraoperative bleeding, prior to surgery, the renal artery was embolized in patient 4.

**Table 2:** Clinical characteristics of the patients

**Characteristics**	**Patient 1**	**Patient 2**	**Patient 3**	**Patient 4**
Sex	Male	Male	Male	Male
Age (y)	68	61	43	64
Active smoking	Yes	Yes	Yes	Yes
Other personal history	No	No	No	No
Symptoms	Lower limb edema, back pain	Dyspnea and pulmonary embolism	Right hypochondrium pain	Lower limb edema, increased abdominal girth and itching
Pathological anatomy	Hepatocarcinoma	Adrenal cell carcinoma	Adrenal cell carcinoma	Clear cell renal carcinoma
Tumor stage	T4M1	T4	T4N1	T3b
Tumor thrombus extension	Right atrium	Right atrium Level IV	Right atrium Level IV	Right atrium Level IV
Preoperative renal artery embolization	Not applicable	Not applicable	Not applicable	Yes

Table 3 shows the surgical details and the evolution and follow-up of each case.

**Table 3:** Surgical data and hospital stay (ECC: extracorporeal circulation, CFV: common femoral vein, SVC: superior vena cava)

	**Patient 1**	**Patient 2**	**Patient 3**	**Patient 4**
ECC time (min)	160	101	54 • Veno-venous bypass: 30 min • ECC: 24 min	59
Aortic clamping time (min)	53	69	0	0
Circulatory arrest time (min)	0	0	0	0
Hepatic ischemia time (min)		Pringle maneuver not performed	31	20
Aortic cannulation	Ascending aorta	Ascending aorta	Ascending aorta	Brachiocephalic trunk
Venous cannulation	SVC and CFV	SVC and CFV	SVC and CFV	SVC and CFV
IVC reconstruction with pericardial patch	No	No	Yes	Yes
Time in ICU (days)	3	1	8	3
Hospital stay (days)	8	17	16	17
Postoperative complications	No	No	Minimal liver dysfunction	Atrial fibrillation
Hospital mortality	No	No	No	No
Follow-up mortality	Yes	Yes	No	No
Cause of death	Disease progression	Disease progression	Alive	Alive
Follow-up time (months)	7.5	17.4	96.1	2.4

*ECC: extracorporeal circulation, CFV: common femoral vein, SVC: superior vena cava*

 The mean bypass time was 93.5 min (54-160). During the intervention, circulatory arrest was not necessary in any case. In the third case, we performed a veno-venous bypass (V-V) with the intention of a less invasive approach. During the resection of the tumor thrombus, the patient presented significant venous bleeding that required conversion to cardio-pulmonary bypass in order to hemodynamically stabilize the patient.

 In the intervention of the fourth patient, an appendectomy was performed, discovering in the pathological anatomy a low-grade appendiceal mucinous neoplasm. Aortic cannulation of the BCT was performed in order to use it for antegrade cerebral protection in case of hypothermic circulatory arrest, which might be needed in the event of excessive bleeding in the surgical field.

 None of the patients died during their hospital stay or within 30 days. We found two postoperative complications: mild hepatic dysfunction due to the Pringle maneuver and the corresponding intraoperative hepatic ischemia; and one episode of atrial fibrillation resolved before hospital discharge.

 The mean follow-up time was 30.85 months (2.4-96.1). The clinical and radiological follow-up consisted of reviews and serial imaging tests (CT) at 1 month, 3 months and according to individual requirements and the patient’s condition by the Oncology service.

 Currently, two patients are still alive and two have died due to disease progression. One of the two patients under follow-up presented tumor recurrence due to adrenal carcinoma metastasis in the upper lobe of the right lung, requiring lobectomy and adjuvant treatment.

## Discussion

Tumor thrombi that invade the vena cava classically present with symptoms of lumbar pain, abdominal mass, hematuria, and anemia, among others.^2,3^ In these cases, the only curative treatment is surgery by total resection of the tumor and complete removal of the thrombus.

 Our series of cases covers a very long period of time; however, we observe that we have made almost no changes in terms of surgical strategy.

Regarding preoperative renal artery embolization to prevent bleeding performed in the last patient operated on in their meta-analysis, Lardas et al. did not find a clear benefit in it.^8^

 In this same meta-analysis, ECC to treat RCC with level IV tumor thrombus is associated with a shorter bypass and total intervention time compared to deep circulatory arrest. However, in our experience, circulatory arrest was not necessary in any case, but we defend that it is a good strategy to avoid the damage derived from hot hepatic ischemia, and achieve a completely bloodless surgical field; in contrast, it increases neurological morbidity and coagulation disorders derived from thrombocytopenia and platelet dysfunction. Whether with ECC or circulatory arrest, these two strategies must be taken into account when planning the intervention; without them; the risk of intraoperative bleeding is deemed unacceptable. In this aspect, we should highlight the importance of the cell-saver technique, it is a cost-effective and safe method of autologous blood reinfusion, feasible in any cardiac intervention, without causing cardiovascular, neurological, and immunological adverse effects. Cell salvage requires no preoperative preparation of the patient, making it ideal for unexpected massive hemorrhage, but it contains no platelets or coagulation factors; so, in such cases, the patient will require allogeneic blood components.

In their multicenter study, Abel et al. collected 162 patients with RCC and tumor thrombus of levels III and IV. They describe an incidence of serious complications such as postoperative bleeding, cardiac arrest, or thromboembolism of 34%. In their analysis, they observe that these events are associated with risk factors such as tumor stage and the level of the tumor thrombus, among others.^9^ The risk of hospital mortality varies between 1% and 10% depending on the series, and according to the experience of the center.^9,10^

In the case of RCC, 5 years after surgery, the estimated survival is 40-63%, and the disease-free percentage is 35-55% depending on the series, and it depends on the tumor stage and not on the level of the thrombus.^9^

Regarding the level of tumor thrombosis, the presentation of level IV thrombus implies a higher risk of short-term complications but does not affect survival compared to thrombi of other lower levels (levels I-III).^8^

 Our study has some limitations. The number of cases is small, with the first and last being temporarily spaced 12 years apart, and the follow-up time is short. On the other hand, data has been collected retrospectively.

## Conclusion

 We present a series of patients who underwent lumpectomy and IVC thrombectomy, who’s postoperative and mid-term results are encouraging. Tumor thrombosis of the IVC has an ominous prognosis, however surgical treatment plays an important role by improving the survival of these patients. The level of thrombosis is not a risk factor for long-term survival; as is adversely the stage of the tumor. A multidisciplinary approach is necessary to obtain good postoperative results.

## Competing Interests

 None declared.

## Ethical Approval

 Approval of the Local Ethics Committee of Álvaro Cunqueiro Hospital of Vigo was obtained by following the dictated requisite of waived informed consent of the patients.

## Funding

 No funding was received for this work.
